# Trauma-triggered coronary embolism causing extensive anterolateral ST-segment elevation myocardial infarction in a young man with a mechanical aortic valve: a case report

**DOI:** 10.1093/ehjcr/ytag381

**Published:** 2026-06-04

**Authors:** Davar Aledavood, Zahra Alizadeh, Mahsa Borjzadehgashtaseb

**Affiliations:** Department of Cardiology, Shiraz University of Medical Sciences, Shiraz 7134814336, Iran; Department of Cardiology, Shiraz University of Medical Sciences, Shiraz 7134814336, Iran; Department of Cardiology, Shiraz University of Medical Sciences, Shiraz 7134814336, Iran

**Keywords:** Blunt chest trauma, Coronary embolism, Mechanical aortic valve, Mural thrombus, ST-segment elevation myocardial infarction, Tirofiban, Apical aneurysm, Embolic thrombus

## Abstract

**Background:**

Coronary embolism is a rare but clinically important non-atherosclerotic cause of acute myocardial infarction. Its diagnosis becomes especially challenging in young patients with mechanical valves, especially when the presentation follows blunt chest trauma.

**Case summary:**

A 24-year-old man with a mechanical aortic valve presented with progressive chest pain and dyspnoea 2 days after blunt chest trauma. Electrocardiography (ECG) demonstrated extensive anterior–lateral ST-segment elevation myocardial infarction (STEMI). Transoesophageal echocardiography revealed normal prosthetic valve function, an apical aneurysm, and a non-mobile left ventricular (LV) mural thrombus. Coronary angiography showed a large thrombotic occlusion in the proximal left anterior descending (LAD) artery with otherwise normal coronary anatomy. Findings were most consistent with coronary embolism originating from a trauma-induced LV mural thrombus. Given the high thrombus burden, the patient received dual antiplatelet therapy, continued warfarin, and tirofiban infusion. Follow-up imaging confirmed complete resolution of the thrombus and improvement in ventricular function.

**Discussion:**

This case illustrates the rare mechanism of trauma-induced LV mural thrombus embolization causing STEMI in a young prosthetic-valve patient. Integration of multimodality imaging coupled with meticulous clinical reasoning is required for systematic exclusion of spontaneous coronary artery dissection (SCAD), infective endocarditis, prosthetic-valve thrombosis, hypercoagulable states, and *in situ* coronary thrombosis to establish the true aetiology. Tirofiban may be an effective adjunct in high-burden embolic coronary occlusions when distal flow is preserved.

Learning points

- Coronary embolism should be considered in young patients presenting with ST-segment elevation myocardial infarction (STEMI), particularly when there is a recent history of blunt chest trauma.
- Blunt chest trauma can trigger left ventricular mural thrombus formation and subsequent coronary embolism, even in well-anticoagulated patients.
- Multimodality imaging combined with careful exclusion of differentials is essential for confirming an embolic aetiology in trauma-associated STEMI.

## Introduction

Myocardial infarction in a young adult without traditional cardiovascular risk factors requires consideration of non-atherosclerotic causes.^1^ Coronary embolism is an increasingly recognized but often underdiagnosed cause of acute coronary syndromes.^2^ Mechanical aortic valves predispose to thrombosis due to non-physiological flow patterns, continuous shear stress, and platelet activation.^3^

Blunt chest trauma is an infrequent precipitating factor for myocardial infarction,^4^ but it may lead to myocardial contusion, regional akinesia, apical aneurysm, and localized stasis—all conditions that predispose a patient to thrombus formation.^5^ The combination of prosthetic valve thrombogenicity and trauma-induced myocardial insult leading to coronary embolism represents a truly unusual clinical event. This case provides the opportunity for in-depth consideration of this interaction and of the differential diagnosis that must be performed.

## Summary figure

**Figure:**
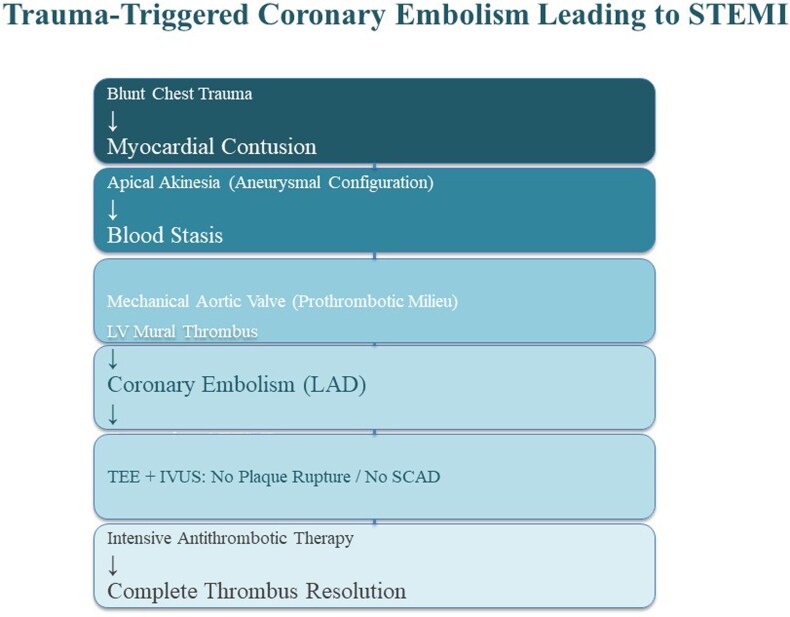


## Case presentation

A 24-year-old man with a history of mechanical aortic valve replacement (AVR) performed 10 years ago for congenital aortic stenosis had no significant family history of cardiovascular disease. He had no history of smoking or alcohol use. He weighed 70 kg and was 175 cm tall (body mass index 22.9 kg/m^2^). He was on warfarin 5 mg daily and aspirin 80 mg daily, with a stable international normalized ratio (INR) of around 2.5 during routine follow-ups. He was fully functional with a stable clinical status before the event.

Two days before admission, following a blunt chest trauma while playing football, he developed progressive retrosternal chest pain, cold sweating, and dyspnoea, without nausea or vomiting. On presentation, he was alert and haemodynamically stable, with a blood pressure of 110/80 mmHg, heart rate of 66 b.p.m., respiratory rate of 18/min, temperature of 36.2°C, and oxygen saturation of 96% on room air. Cardiac examination revealed an audible prosthetic valve click without additional abnormal heart sounds. Peripheral pulses were symmetric. There were no signs of pulmonary oedema or other respiratory abnormalities.

Initial laboratory tests showed a white blood cell count of 9.4 × 10^9^/L (normal range: 4–10 × 10^9^/L), haemoglobin level of 16.8 g/dL (normal range: 13–17 g/dL), and platelet count of 203 × 10^9^/L (normal range: 150–400 × 10^9^/L). Electrolytes and renal function were normal. The INR on admission was 2.5 and remained within this therapeutic range throughout hospitalization.

The initial electrocardiogram (ECG) demonstrated sinus rhythm, ST-segment elevation accompanied by Q waves and T-wave inversion in leads V1–V6, I, and aVL, along with reciprocal ST-segment depression in the inferior leads and aVR, consistent with an anterolateral ST-segment elevation myocardial infarction (STEMI) (*Figure 1*). The patient was admitted to the coronary care unit and received initial treatment, including aspirin 325 mg loading, clopidogrel 300 mg loading followed by maintenance doses, rosuvastatin 40 mg daily, bisoprolol 2.5 mg daily, captopril 6.25 mg twice daily, and morphine for pain control. Warfarin therapy was continued.

**Figure 1:**
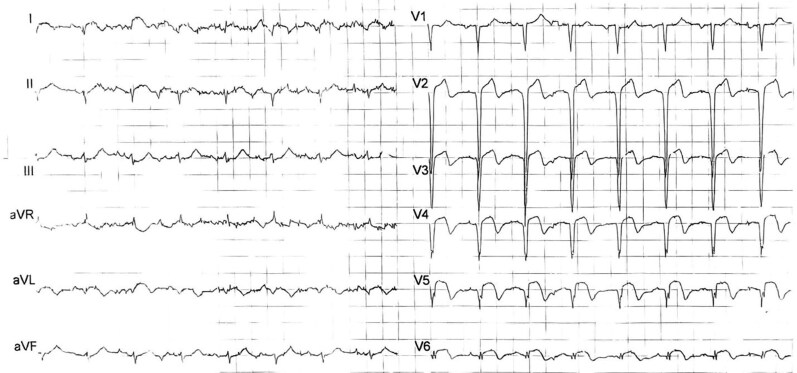
The first electrocardiogram shows sinus rhythm, ST-segment elevation accompanied by Q waves and T-wave inversion in leads V1–V6, I, and aVL, along with reciprocal ST-segment depression in the inferior leads and aVR, consistent with an anterolateral ST-segment elevation myocardial infarction.

Given his young age, previous AVR, and trauma-related onset, differential diagnoses included prosthetic valve dysfunction, valve-related embolism, trauma-induced coronary injury, spontaneous coronary artery dissection, and premature atherosclerosis.

Transoesophageal echocardiography (TEE) showed a normal left ventricular (LV) size with mild systolic dysfunction (left ventricular ejection fraction 40%), akinesia of the apical and anterior walls with apical aneurysm, and a non-mobile apical mural thrombus measuring 8 × 2.2 mm. The mechanical aortic valve demonstrated normal leaflet motion and haemodynamics [mean pressure gradient 18.3 mmHg, acceleration time 74 ms, Doppler velocity index (DVI) 0.29, *V*_max_ 3.58 m/s], with no thrombus or pannus.

The patient was transferred to the catheterization laboratory, where fluoroscopy confirmed normal prosthetic valve motion. Coronary angiography revealed a large thrombotic lesion and occlusion in the late proximal left anterior descending (LAD) artery, with ostial stenosis of the diagonal branch; other vessels were normal (*Figure 2*).

**Figure 2:**
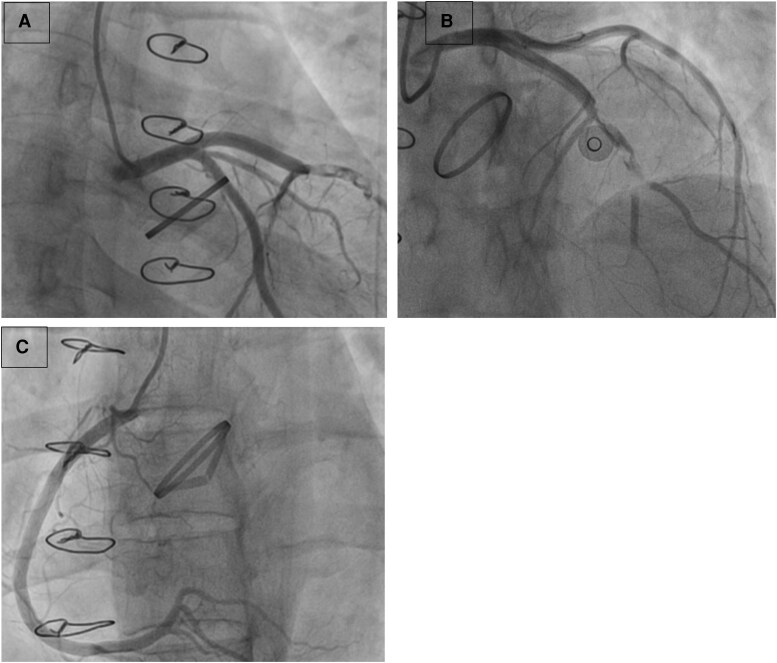
First coronary angiography. (*A*) Right anterior oblique (RAO)-caudal projection showing normal left main and left circumflex arteries, with occlusion of the left anterior descending artery due to a large thrombotic lesion. (*B*) Right anterior oblique-cranial projection showing normal left main and left circumflex arteries, with occlusion of the left anterior descending artery due to a large thrombotic lesion, along with ostial stenosis of the diagonal branch. (*C*) Left anterior oblique (LAO) projection showing a normal right coronary artery and its branches.

Intravascular ultrasound (IVUS) was performed and showed a normal endothelial layer without evidence of spontaneous coronary artery dissection or plaque rupture. The findings supported an embolic aetiology rather than structural coronary pathology. Given preserved distal thrombolysis in myocardial infarction (TIMI) flow and embolic morphology, mechanical intervention was deferred to avoid distal embolization. The patient received tirofiban infusion (30 mL over 5 min followed by 10 mL/h for 18 h). The use of tirofiban in the context of post-traumatic embolic STEMI in a patient with a mechanical valve has not been widely reported. Follow-up echocardiography showed no significant change. He improved clinically with resolution of chest pain and dyspnoea and was discharged on triple therapy.

At 1-month follow-up, INR was 2.8, ejection fraction (EF) had improved to 45%, and the apical thrombus had decreased in size. Repeat coronary angiography and IVUS revealed normal coronary arteries with no residual thrombus, and the patient continued on warfarin monotherapy (*Figures 3* and *4*).

**Figure 3:**
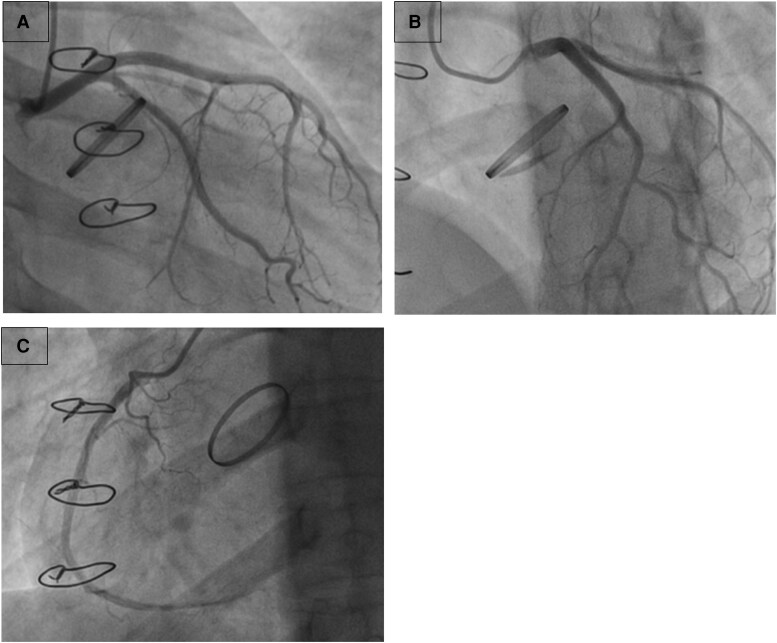
Final coronary angiography after medical treatment with antiplatelet and anticoagulant therapy. (*A*) Right anterior oblique (RAO)-caudal projection and (*B*) left anterior oblique (LAO)-cranial projection showing normal left coronary system arteries. (*C*) Left anterior oblique projection showing a normal right coronary artery and its branches.

**Figure 4:**
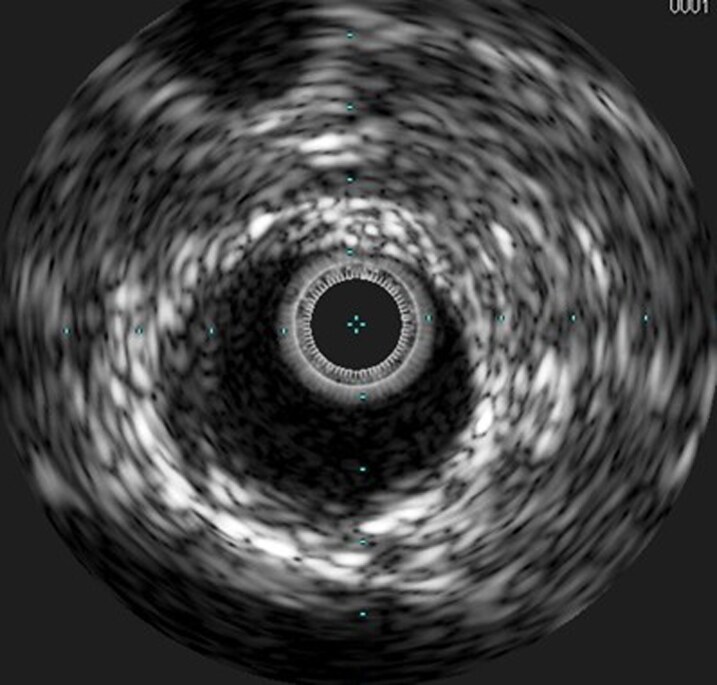
Final intravascular ultrasound after antiplatelet and anticoagulant therapy, showing normal findings.

This case highlights a rare occurrence of post-traumatic embolic STEMI in a young patient with a mechanical aortic valve, complicated by apical and anterior LV thrombus.

## Discussion

This case illustrates a rare but coherent pathophysiologic sequence wherein blunt chest trauma precipitates myocardial contusion, is followed by apical aneurysm formation, regional akinesia, blood stasis, and mural thrombus development.^6^ Even therapeutic anticoagulation may not always fully prevent thrombosis in the inherently thrombogenic environment of a mechanical valve.^7^ Embolization of the LV thrombus into the LAD culminated in extensive anterior–lateral STEMI. The patient’s young age, absence of cardiovascular risk factors, angiographically normal coronaries except for the thrombotic occlusion, and presence of LV thrombus and normal prosthetic valve function significantly enhanced diagnostic confidence. These features distinctly differentiated the event from plaque-mediated STEMI or spontaneous coronary artery dissection. Trauma served as a key initiating factor by precipitating local stasis and regional dysfunction because of myocardial injury.^5^ The development of LV aneurysm and mural thrombus shortly after chest impact strongly suggests that trauma was a proximal initiating factor.

## Differential diagnosis

The differential diagnosis of anterior STEMI in a young patient with recent blunt chest trauma and a mechanical aortic valve requires a systematic and mechanistic approach. Spontaneous coronary artery dissection (SCAD) was initially considered, as it is a recognized cause of myocardial infarction in younger individuals. However, the absence of typical angiographic or IVUS features—such as a dissection flap, intramural haematoma, or vessel wall irregularity—argued against this diagnosis.^8^ Atherosclerotic plaque rupture or erosion was similarly unlikely given the completely normal coronary anatomy and absence of traditional cardiovascular risk factors. Infective endocarditis, although relevant in the setting of a prosthetic valve, was excluded based on the absence of clinical features and the lack of vegetations or prosthetic dysfunction on TEE.

*In situ* thrombosis of the LAD was considered but did not match the imaging profile. This mechanism typically involves plaque disruption or endothelial injury, neither of which was present.^9^ Intravascular ultrasound demonstrated an intact vessel wall with no evidence of underlying disease or trauma, strongly supporting an embolic aetiology. Systemic hypercoagulability and warfarin resistance were also assessed. The therapeutic INR at presentation, lack of prior INR instability, and absence of clinical features of thrombophilia made these explanations unlikely.^7,10,11^ Instead, thrombus formation in an area of trauma-related apical akinesia provides a more coherent explanation, as low-flow regions may form thrombus despite therapeutic anticoagulation.

Myocardial contusion was a relevant competing diagnosis given the recent blunt trauma. Although cardiac magnetic resonance imaging (MRI) is the most sensitive modality for identifying contusional injury, it was not feasible in the acute setting due to the patient’s haemodynamic instability and need for urgent reperfusion and limited availability of urgent cardiac MRI at our centre. This remains a limitation. Nonetheless, multimodality imaging offered important diagnostic clarity. Transoesophageal echocardiography identified an apical aneurysmal segment with an adherent mural thrombus, and angiography revealed a large thrombus within a normal proximal LAD. Intravascular ultrasound further confirmed preserved vessel wall integrity. Taken together, these findings did not support myocardial contusion as the primary mechanism of myocardial injury.

Stress cardiomyopathy (Takotsubo syndrome) was also considered. However, several key features weighed against this diagnosis. The patient was a young male, whereas Takotsubo syndrome predominantly affects post-menopausal women. Moreover, the presence of an obstructive proximal LAD thrombus is incompatible with the non-obstructive pattern characteristic of Takotsubo syndrome.

Integrating the clinical context and imaging findings, embolization from a trauma-induced LV mural thrombus represents the most plausible mechanism. The sequence of blunt chest trauma, development of apical wall motion abnormality, subsequent mural thrombus formation in a low-flow region, and eventual embolization into a structurally normal LAD provides a coherent explanation for the presentation.^12^

Management required careful balancing of thrombus resolution with bleeding risk in a patient already receiving warfarin. Avoiding catheter-based manipulation was considered in the context of embolic morphology and preserved distal flow, to reduce the risk of distal embolization. A pharmacologic approach was therefore selected. Short-term tirofiban in combination with dual antiplatelet therapy and continued anticoagulation provided effective thrombus dissolution without complications. The addition of tirofiban to ongoing warfarin and dual antiplatelet therapy increased the theoretical bleeding risk; however, the infusion was limited to 18 h, the patient remained haemodynamically stable with no signs of bleeding, and close monitoring of haemoglobin and clinical status was performed. The decision was justified by the high thrombus burden and the goal of rapid restoration of coronary flow without mechanical manipulation in a young patient with otherwise normal coronaries. Follow-up imaging confirmed complete resolution. This experience suggests that in selected cases of embolic STEMI with preserved downstream flow, a conservative medical strategy may be both safe and effective.

This case highlights the diagnostic challenges of trauma-related myocardial infarction and demonstrates the value of multimodality imaging—particularly IVUS and TEE—in distinguishing embolic from traumatic or atherosclerotic mechanisms. It also underscores that in appropriately selected patients, conservative medical therapy may be a reasonable alternative to mechanical intervention in embolic STEMI.

## Conclusion

This case illustrates the unusual interplay of blunt chest trauma, prosthetic valve physiology, and LV mural thrombus formation culminating in coronary embolism and extensive STEMI. A systematic approach was necessary that excluded SCAD, infective endocarditis, prosthetic valve embolism, hypercoagulability, *in situ* thrombosis, and atherosclerotic disease. Tirofiban was useful in accomplishing complete thrombus resolution.

## Limitations

Despite the clinical trajectory and multimodality imaging that strongly support trauma-induced mural thrombus embolization, other investigations, such as cardiac MRI, might have given more definite evidence of myocardial contusion or microvascular injury. Thrombophilia testing was not performed, given the clear traumatic trigger and therapeutic INR; hence, an underlying hypercoagulable predisposition cannot be fully excluded.

## Data Availability

All data supporting the findings of this case report are contained within the manuscript and the accompanying figures. No additional datasets were generated or analysed.

## Lead author biography

Dr Davar Aledavood is a cardiologist and cardiovascular specialist currently serving as an attending physician in Shiraz, Iran. He is dedicated to the prevention, diagnosis, and treatment of heart and vascular diseases, with a strong commitment to evidence-based medicine and patient-centred care. Dr Aledavood has experience managing a wide range of cardiac conditions, including coronary artery disease, heart failure, arrhythmias, and hypertension. Known for his clinical precision and compassionate approach, he strives to provide comprehensive cardiovascular care tailored to each patient’s individual needs while promoting long-term heart health and improved quality of life.

## Notes

**Consent:** All authors are fully satisfied with the publication of the article. The authors confirm that written consent for submission and publication of this case report, including images and associated text, has been obtained from the patient in line with the COPE guidelines.
